# Berberine-Loaded Biomimetic Nanoparticles Attenuate Inflammation of Experimental Allergic Asthma *via* Enhancing IL-12 Expression

**DOI:** 10.3389/fphar.2021.724525

**Published:** 2021-11-09

**Authors:** Hua Jin, Jiale Li, Miaoyuan Zhang, Renxing Luo, Peishan Lu, Wenting Zhang, Junai Zhang, Jiang Pi, Weixin Zheng, Zesen Mai, Xiaowen Ding, Xinguang Liu, Suidong Ouyang, Gonghua Huang

**Affiliations:** ^1^ Guangdong Provincial Key Laboratory of Medical Molecular Diagnostics, Guangdong Medical University, Dongguan, China; ^2^ College of Pharmacy, Guangdong Medical University, Dongguan, China; ^3^ College of Medical Technology, Guangdong Medical University, Dongguan, China

**Keywords:** berberine, biomimetic nanoparticles, platelet membrane, asthma, IL-12

## Abstract

Asthma is one of the most common chronic pulmonary disorders, affecting more than 330 million people worldwide. Unfortunately, there are still no specific treatments for asthma so far. Therefore, it is very important to develop effective therapeutics and medicines to deal with this intractable disease. Berberine (Ber) has fabulous anti-inflammatory and antibacterial effects, while its low water solubility and bioavailability greatly limit its curative efficiency. To improve the nasal mucosa absorption of poorly water-soluble drugs, such as Ber, we developed a platelet membrane- (PM-) coated nanoparticle (NP) system (PM@Ber-NPs) for targeted delivery of berberine to the inflammatory lungs. *In vivo*, PM@Ber-NPs exhibited enhanced targeting retention in the inflammatory lungs compared with free Ber. In a mouse model of house dust mite- (HDM-) induced asthma, PM@Ber-NPs markedly inhibited lung inflammation, as evident by reduced inflammatory cells and inflammatory cytokines in the lung compared with free Ber. Collectively, our study demonstrated the inhibitory actions of nasally delivered nanomedicines on HDM-induced asthma, primarily through regulating Th1/Th2 balance by enhancing IL-12 expression which could potentially reduce lung inflammation and allergic asthma.

## Introduction

**FIGURE 1:**
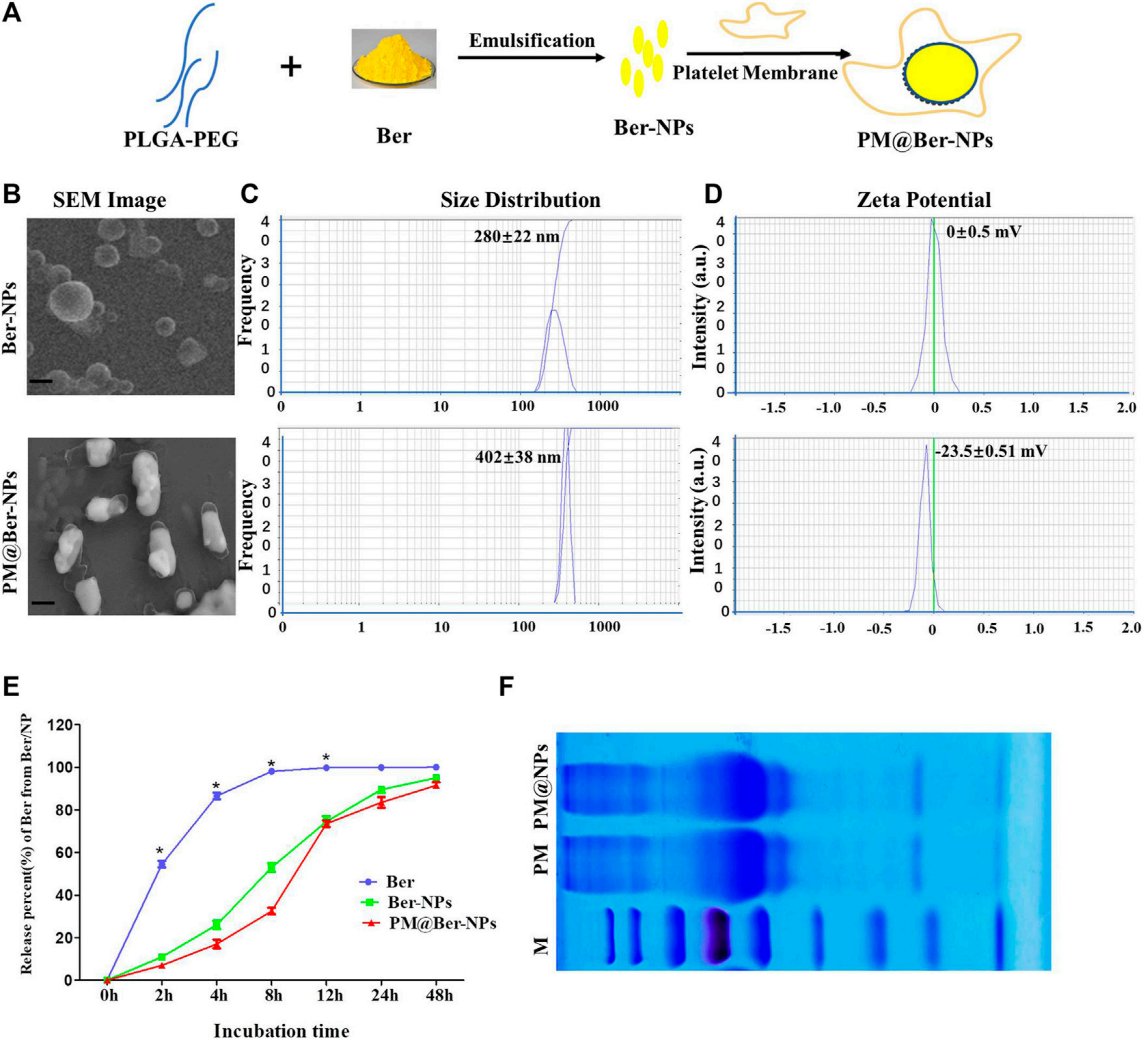
Characterization of Ber-loaded NPs. **(A)** Scheme of preparation of biomimetic-based platelet-cell membrane-coated nanoparticles (PM@Ber-NPs). **(B)** Scanning electron microscope (SEM) image of Ber-NPs and PM@Ber-NPs. Scale bar = 100 nm. **(C)** Mean size and **(D)** zeta potentials of Ber-NPs and PM@Ber-NPs. **(E)** *In vitro* release kinetics of berberine from nanoparticles (NPs) in PBS (0.01 M, PH = 7.4). **(F)** SDS-PAGE protein analysis of platelet membrane (PM) and PM@NPs. *p < 0.05.

As one kind of chronic inflammatory disease of the airway, asthma is still regarded as a great medical and socioeconomic burden worldwide (Barberand et al., 2021; Klimek et al., 2021). In the past decades, asthma treatment is mainly through the inhalation or systemic administration of bronchodilators and anti-inflammatory agents, such as glucocorticoids (Wu et al., 2021). However, it presents major challenges in drug delivery and therapeutic efficacy due to the mucus obstruction in airway inflammation diseases; thus, the pathological symptoms cannot be controlled even with high doses of the recommended drugs (Chakraborty et al., 2021). Besides, chemotherapeutic agents and glucocorticoids always show serious side effects in the body (Cangemi et al., 2021). To address the problems in clinical asthma treatment, we synthesized platelet-derived extracellular vesicles- (PEVs-) cloaked biomimetic nanoparticle (NP) carrier for targeted delivery of natural herbal ingredients berberine (Ber) to the asthma airway, which is expected to alleviate the symptoms and progression of pulmonary inflammatory disease (as shown in Figure 1A).

Berberine is a quaternary ammonium isoquinoline alkaloid, which is extracted from Chinese medicine *Berberis pruinosa*. *Berberis pruinosa* has been used in Chinese traditional medicine to treat infection, diabetes, arrhythmia, tumors, osteoporosis, etc. (Mujtaba et al., 2021; Ye et al., 2021) for thousands of years, which indicates the good biological safety of Ber. However, the therapeutic effects of Ber are greatly limited owing to its poor bioavailability and off-targeting properties.

NP-based delivery systems have been extensively developed to load and deliver Ber to disease sites for enhanced biocompatibility purposes (Zhao et al., 2021). Cell membrane-cloaked biomimetic drug delivery system has recently attracted increasing attention due to endowing NPs with enhanced biocompatibility, low immunogenicity, and active targeting abilities (Vijayan et al., 2018). Platelets are small blood cells (1∼3um) that have superior inflammation targeting properties. However, it might evoke inflammation in the body through directly adopting platelets as drug carriers. To solve this problem, researchers developed a PEV platform. PEVs are nanosized membrane vesicles (100–150 nm) derived from platelets. PEVs-cloaked carriers could avoid phagocytic uptake by macrophages and could target and adhere to injured parts of the vasculature, which can enhance the binding between platelet-adhering pathogens and inflammatory tissues (Ma et al., 2020).

In this work, to address the off-targeting properties of Ber *in vivo*, the biomimetic NPs were synthesized to load and deliver Ber to the inflammatory lungs. The biomimetic NPs could be targeted and retained in the inflammation sites due to the inflammatory targeting characteristics of platelet membrane (PM) which is coated onto the surface of NPs. In this study, we found that Ber-loaded biomimetic NPs could successfully attain inflammatory lungs at 2 h after NPs administration, while the Ber was released from NPs from 2 to 48 h. It implied that NPs could deliver and release the Ber into lungs that play anti-inflammation effects and subsequently attenuate allergic asthma.

## Materials and Methods

### Mice

Eight-week-old female C57BL/6 mice were obtained from Laboratory Animal Center, Southern Medical University (Guangzhou, China). The experimental protocols were conducted according to the National Institutes of Health guidelines on the use of laboratory animals. The animal care and study protocols were approved by the Institutional Animal Care and Use Committee of Guangdong Medical University (GDY2002094).

### Antibodies

Anti-mouse CD11c (N418), MHC-II (M5/114.15.2), CD11b (M1/70), Ly6G (RB6-8C5), SiglecF (E50-2440), CD4 (RM4-5), TCRβ (H57-597), IL-4 (11B11), IL-13 (eBio13A), and IL-12p40 (C17.8) antibodies were obtained from eBioscience. Anti-mouse IL-5 (TRFK5) was obtained from BD Biosciences.

### Cell Culture and NP Uptake

A549 cells were grown in Dulbecco’s Modified Eagle Medium (DMEM, Gibco, Grand Island, NY) supplemented with 10% fetal bovine serum (FBS, Gibco) and 1% penicillin-streptomycin (Gibco) at 37°C in a 5% CO_2_ incubator. In experiments to determine the involvement of NPs uptake, the cells were incubated with 50 μg/mL house dust mite (HDM, *Dermatophagoides farinae,* Greer Laboratories) for 24 h. Then, A549 cells were treated with naked Cou6 NPs or PM@Cou6 NPs for 2 h. After the cells were washed two times, A549 cells treated with or without HDM were imaged by fluorescence microscopy (Olympus IX70 Inverted Microscope).

### Preparation Berberine-Loaded Nanoparticles (Ber-NPs)

Ber-NPs were prepared using emulsification and evaporation methods, as previously described with some modifications (Liu et al., 2010). Briefly, 20 mg of Ber (Sigma-Aldrich) and 80 mg of PEG/PLGA (PEG5000-PLGA28,000, Sigma-Aldrich) were dissolved in 5 mL of dichloromethane as the oil (O) phase, while 20 mL of PVA (1%, w/w, Sigma-Aldrich) was used as external the water (W) phase. The oil phase was sonicated for 40 s at 100 W on ice to form the first emulsification. Then, it was dropped into the W phase and sonicated for another 40 s to form the second emulsification. 250 μg of Coumarin 6 (Cou6, Sigma-Aldrich, United States) was added to the oil phase as the fluorescence marker of NPs. Finally, the NPs were harvested by centrifuging at 12,000 rpm for 20 min and washed three times with water.

### Preparation of PM-Coated Ber-Loaded NPs

Fresh whole blood samples were drawn from healthy mice. Platelets from whole blood were isolated through gradient centrifugation. Briefly, 2.5 mL whole blood (from five mice) was centrifuged at 200 g for 10 min and the supernatant was isolated as platelet-rich plasma (PRP). Then, the PRP was centrifuged at 1800 g for 20 min at 4°C, followed by discarding the supernatant. The platelets were accumulated in the precipitates and were washed twice with PBS before use.

Platelets were suspended in deionized water with protease inhibitor and frozen at −80°C, followed by thawing at room temperature. After three repeated freeze-thaw cycles and sonication, the platelets were mixed with Ber-NPs and sonicated for 30 s.

### Characterization of PM@Ber-NPs

The binding capacity of PM coating on Ber-NPs was calculated by measuring the concentrations of unbound PM in the supernatant and in the wash solution. The entrapment efficiency of Ber by the different types of NPs was assessed by high-performance liquid chromatography (HPLC). The size distribution and zeta potential of the NPs were determined using a Zetasizer Nano ZS (Malvern Instruments, United Kingdom). The morphology of the as-prepared Ber-NPs was captured using the ZEISS scanning electron microscope (SEM) operated at a 15.0 kV accelerating voltage.

To measure the Ber loading rate of Ber-NPs or PM-Ber-NPs, 10 mg lyophilized NPs were dissolved in 1 mL of methanol, and then the amount of Ber in the solution was determined by HPLC. HPLC detection was performed using a C18 column (5 μm, 250 mm × 4.6 mm), whereas the mobile phase, consisting of acetonitrile and phosphate buffer [0.05 mol/L potassium dihydrogen phosphate and 0.05 mol/L sodium heptane sulfonate (1:1)] (40:60), was maintained at a flow rate of 1.0 mL/min. The ultraviolet detector wavelength was 263 nm and the injection volume was 20 μL.

### HDM-Induced Asthma

Eight-week-old female C57BL/6 mice were sensitized intranasally with HDM (200 μg in 50 μL saline per mouse; *Dermatophagoides farina*, Greer Laboratories) on day 0 and day 1. These mice were subsequently challenged (intranasally) with HDM (30 μg in 50 μL saline per mouse) on day 14 for five consecutive days. The HDM + (Ber-NPs) group and the HDM + (PM@Ber-NPs) group of mice were administered intranasally with Ber (2 mg/kg body weight) 1 h before HDM challenge from day 14 to day 21. Bronchoalveolar lavage fluid (BALF), sera, and lung samples were collected 48 h after the last HDM challenge.

### IVIS Imaging

HDM-induced C57BL/6 mouse asthmatic models were intranasally administered with 50 μL of naked PLGA NPs and PM-coated biomimetic NPs (Cou6 was loaded into the NPs as fluorescence marker). For semiquantitative analysis, the image and fluorescence intensities of mice were collected 2 h after intranasal administration of NPs and determined using the Kodak Multi Mode Imaging System.

### Histology

Lung tissues were fixed and processed for hematoxylin and eosin (H&E) staining. Briefly, lung tissues were fixed by 5 min instillation of 10% PBS-buffered formalin through trachea catheterization at a transpulmonary pressure of 15 cm H_2_O and then kept overnight at 4°C with agitation. After paraffin processing, the tissues were cut into semithin 5 µm thickness and stained with H&E for histological analysis.

### Bronchoalveolar Lavage Fluid Collection and Lung Mononuclear Cell Isolation and Flow Cytometry

For BALF collection, lung tissues were lavaged with 1 mL cold PBS for three times, and the supernatant was collected. Lung mononuclear cells were prepared as previously described (Han et al., 2018). Briefly, lung tissues were removed, minced, and digested with 1 mg/mL collagenase IV (Life Technologies) in RPMI-1640 (Hyclone) with 5% FCS (Hyclone) for 45 min at 37°C. Cells were enriched by using a 38% Percoll gradient (GE Healthcare Life Sciences). Red blood cells were lysed with ACK lysis buffer (R&D Systems). Cells were harvested for analyses. For surface staining, cells were stained with antibodies in PBS containing 1% FCS (Hyclone) on ice for 30 min. For intracellular staining, cells were stimulated with PMA (Sigma-Aldrich) and ionomycin (Sigma-Aldrich) for 5 h in the presence of Golgistop (BD Biosciences) before being stained according to the manufacturer’s instructions (eBioscience). The samples were acquired on a FACSCantoII (BD) or LSRFortessaTM X-20 (BD) and analyzed with FlowJo software (Treestar).

### Blood Collection and ELISA

Blood was collected from the mouse heart and centrifuged at 5000 g at 4°C for 10 min. After being aspirated to blood supernatant, the sera were obtained for ELISA and stored at −20°C. The concentrations of IL-4, IL-5, IL-13, and IL-12 in blood sera were determined by ELISA kits (Invitrogen), according to the manufacturer’s protocol. The linear range of the detection was 0–25,000 pg/mL.

### Real-Time RT-PCR Analysis

To determine the expression of mRNA in lungs, lungs from asthmatic mice were lysed with a tissue homogenizer in TRIzol (Invitrogen). Total RNA was extracted by TRIzol according to the manufacturer’s instructions. Real-time PCR analysis was performed with primers using Power SYBR Green Master Mix from Life Technologies. All gene expression results (mRNA abundances) were expressed as arbitrary units relative to the abundance of GADPH mRNA. The fold change was calculated through the 2-^ΔΔCt^ method. The primers used were as follows: IL-4, forward primer: 5′-GGT​CTC​AAC​CCC​CAG​CTA​GT, reverse primer: 5′-GCC GAT​GAT​CTC​TCT​CAA​GTG​AT; IL-13, forward primer: 5′-CCTGGCTCTTGCTG CCTT, reverse primer: 5′-GGT​CTT​GTG​TGA​TGT​TGC​TCA; IL-5, forward primer: 5′-CTC​TGT​TGA​CAA​GCA​ATG​AGA​CG, reverse primer: 5′-TCTTCAGTATGTCT AGCCCCTG; IL-12, forward primer: 5′-CAA​CCA​TCA​GCA​GAT​CAT​TCT​A, reverse primer: 5′-GAGTCCA GTCCACCTCTACAAC.

### Statistical Analysis

Results are expressed as mean ± SD. Statistical significance was determined by one-way ANOVA with a Games-Howell post hoc analysis for multiple-group comparisons. Two-group comparisons were analyzed by the two-tailed unpaired Student *t*-test.

## Results

### Preparation and Characterization of Ber-NPs and PM@Ber-NPs

To improve the water solubility and bioavailability of Ber, biodegradable polymer PLGA was employed to encapsulate Ber to form soluble NP carriers, and for further increasing the targeting and biocompatibility of NPs, PM-derived vesicles were coated onto the surface of NPs (Figure 1A). The morphology (Figure 1B) and size distribution (Figure 1C) of the obtained Ber-NPs was approximately 280 nm which increased to 400 nm after coating with the cell membrane. According to the SEM results, we assumed that the increased size of NPs might be due to the PM-derived vesicles coating on the surface of NPs. Moreover, the polydispersity of PM@Ber-NPs was <0.3, indicating the uniform dispersion NPs in water.

There are two sides of the cell membrane: one side is with a positive charge and the other side is with a negative charge; the vesicles from the platelet-cell membrane could be coated onto the surface through electrostatic interactions under strong mechanical force. The surface charge (zeta potential) of Ber-NPs was ∼0 mV (Figure 1D), while it increased to −23 mV when coated with PM, which indicates that the PM-derived vesicles have been successfully coated on the surface of NPs. The loading rate of Ber into the NPs was 8.1%, and the encapsulation rate was 81.02%, respectively, as determined by the HPLC method.

### *In Vitro* Release Kinetics of Berberine From NP-Based Delivery System

The *in vitro* release of Ber in its free form was almost completely released within 4 h (98%), with no further increase after 6 h (99%, Figure 1E). Release of Ber from all two types of NPs at 6 h was only 50–60%, with subsequent progressive increases in release at 12 h (65–75%), 24 h (75–85%), and 48 h (90–98%). These data suggested that loading of Ber into NP carriers could delay the release of Ber compared with free berberine and facilitated steady drug release for approximately 24 h.

In addition, SDS-PAGE was performed on PM and PM@NPs (Figure 1F), which indicated that PM@NPs contained endogenous membrane proteins preserved by PM. Our results indicated that the vesicles from platelets were successfully coated or conjugated on the surface of the NPs.

### Enhanced Uptake of PM@Ber-NPs in Inflamed Epithelial Cells Induced by HDM

**FIGURE 2:**
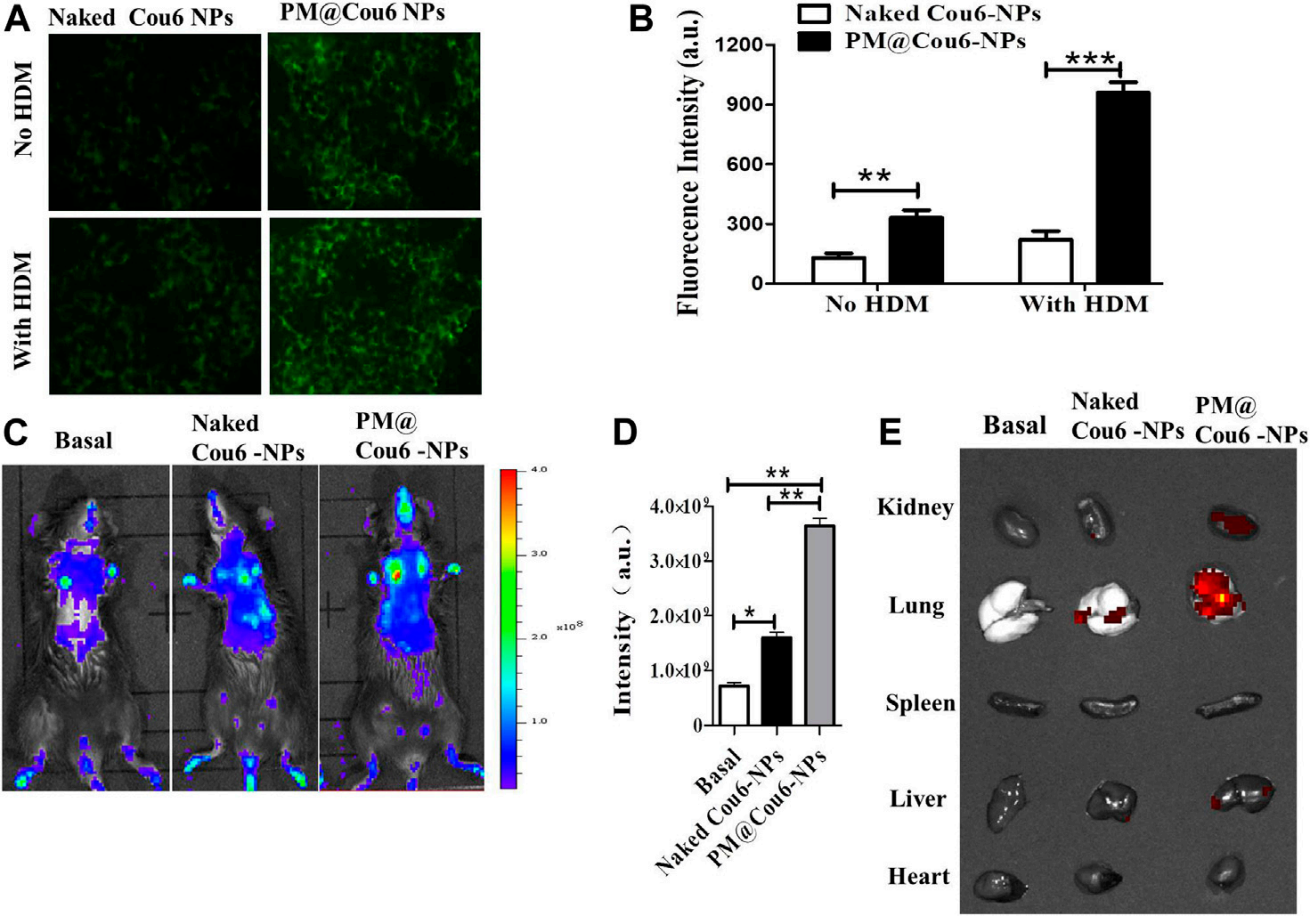
Enhanced retention of drugs in murine inflammatory lungs *via* biomimetic modification of NPs with PM. Cou6 was encapsulated into the NPs as a fluorescence marker. **(A)** The intracellular uptake of Cou6-NPs and PM@Cou6-NPs in A549 cells with or without HDM treatment. **(B)** The fluorescence intensity in A549 cells cocultured with Cou6-NPs and PM@Cou6-NPs. **(C)** Biodistribution of naked Cou6-NPs without modification and PM@Cou6-NPs, determined by the IVIS imaging system. **(D)** The mean fluorescence intensity (MFI) of NPs in the lungs, determined by the IVIS imaging system (*n* = 3). **(E)** Biodistribution and retention of PM@blank-NPs, naked Cou6-NPs without modification, and PM@Cou6-NPs in organs, determined by IVIS imaging system at 2 h after NP intranasal administration. *p < 0.05; **p < 0.01.

To determine whether the biomimetic NPs could target the pulmonary epithelial cells at inflammatory stimulation, we treated A549 cells with HDM for 24 h. As shown in Figures 2A,B, we coincubated the different NP systems with rested or inflammatory (HDM-stimulated) A549 cells for 1 h. In rested A549 cells, NPs uptake represented no significant difference between PLGA NPs and PM@NPs; however, it showed significantly increased cell uptake of PM@NPs in HDM-induced inflammatory A549 cells comparing with PLGA NPs without PM modification. These results demonstrated that PM@NPs could target the inflammatory pulmonary epithelial cells, thus showing higher biocompatibility *in vitro*.

### *In vivo* Biodistribution of NPs in Asthma Mouse Model

*In vivo* and *ex vivo* fluorescence imaging was used to evaluate the pulmonary inflammation targeting capability of Cou6-labeled PLGA NPs (Cou6-NPs) and Cou6-labeled PM@ PLGA NPs (PM@Cou6-NPs). Strong fluorescence signals were observed at lung tissues 2 h after administration with NPs (Figure 2C), demonstrating the enhanced targeting ability of the biomimetic NPs. The statistical data by quantitative region-of-interest (ROI) analysis (Figure 2D) also showed that the fluorescence intensity of lungs of PM@Cou6-NPs was much higher than those of the Cou6-NPs group, indicating that PM@Cou6-NPs had good inflammation targeting properties. In order to investigate which organ PM@Cou6-NPs accumulate in or whether PM@Cou6-NPs pass blood-brain barrier, organs of mice with Cou6-NPs and PM@Cou6-NPs treatments were separated. The results showed that PM@Cou6-NPs mainly accumulate in the lung (Figure 2E; Supplementary Figure S1).

### Ber-Loaded Biomimetic NPs Reduce Cell Infiltration of Airway in HDM-Induced Asthma Mouse

**FIGURE 3:**
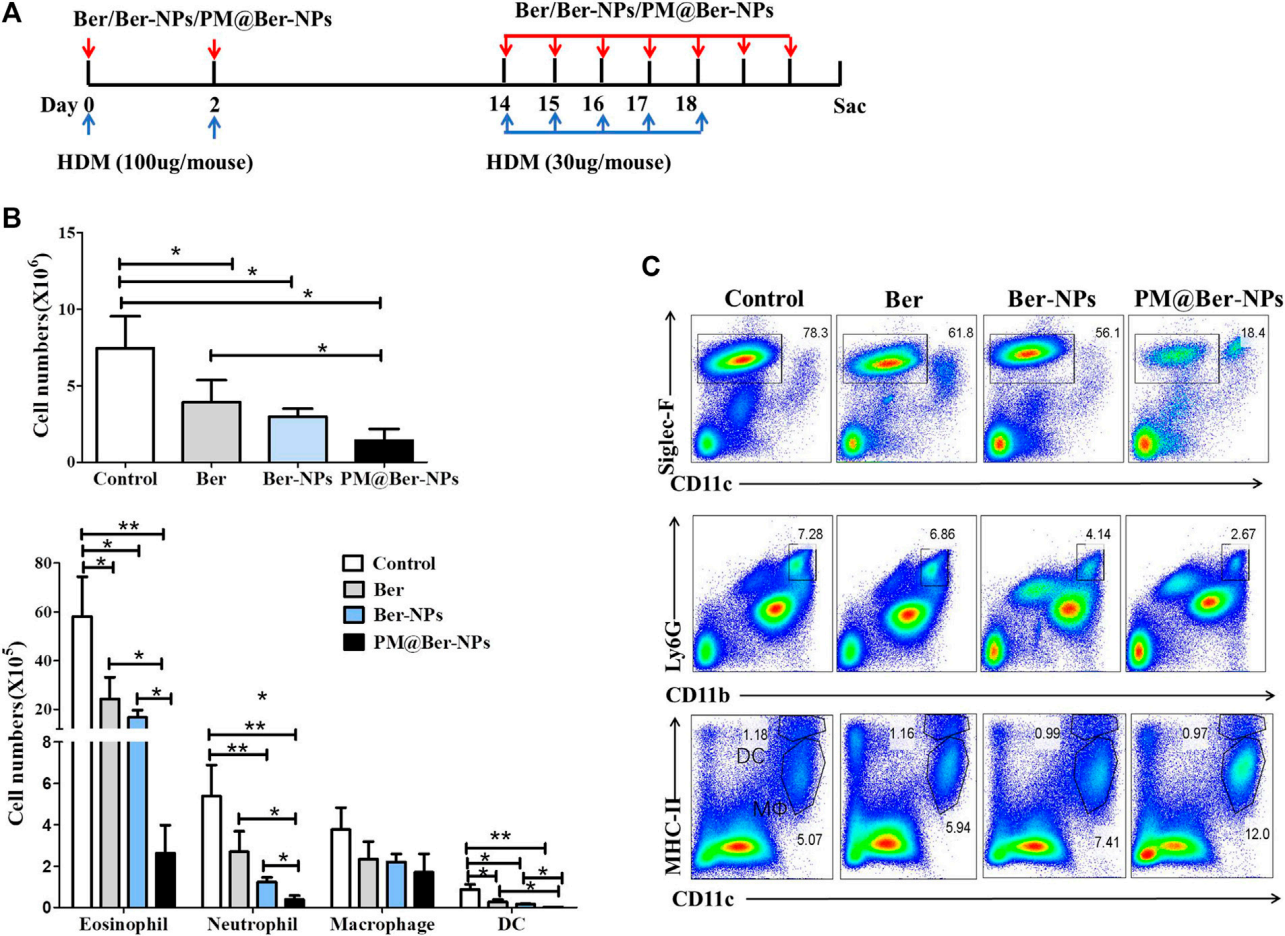
Ber-loaded biomimetic NPs reduce airway cells infiltration in HDM-induced mouse asthma. **(A)** Eight-week-old C57BL/6 female mice (*n* = 5 mice per group) were subjected to the HDM-induced asthma model. PBS (Control), Ber, Ber-NPs, and PM@Ber-NPs were administered to the mice (as described in Methods). Two days after the last challenge, cells in the BALF sera and lungs were collected to further analysis. **(B)** Total cell counts, eosinophils, neutrophils, macrophages, and dendritic cells in the BALF were quantified. **(C)** Infiltrated cells in the BALF with HDM-induced asthma (*n* = 5) were isolated and followed by flow cytometry analysis. *p < 0.05; **p < 0.01.

To investigate the efficacy and specificity of Ber and its NPs *in vivo*, we performed experiments in HDM-mediated eosinophilic airway inflammation. In this HDM-induced asthma model, we treated the mice with Ber and its NPs, during the sensitization and challenge stage of this chronic model (Figure 3A). As shown in Figure 3B, Ber and its NPs reduced total infiltrated cells numbers in BALF of HDM-induced asthmatic mice. Among them, PM@Ber-NPs were the least in reducing cell infiltration into mouse airway. Moreover, flow cytometry showed that Ber and its NPs reduced not only total eosinophil numbers but also total neutrophil numbers and dendritic cells numbers in BALF. But Ber did not affect total macrophage (MΦ) numbers in BALF (Figures 3B,C). Notably, PM@Ber-NPs could reduce much more infiltrated cells in the airway than that of Ber and Ber-NPs. These data indicated that Ber and its NPs inhibited airway inflammation in the HDM-induced asthma model. Additionally, PM@Ber-NPs exhibited the best efficacy to reduce airway inflammation.

### Ber-Loaded Biomimetic NPs Alleviate Lung Inflammation of Asthmatic Mouse

**FIGURE 4:**
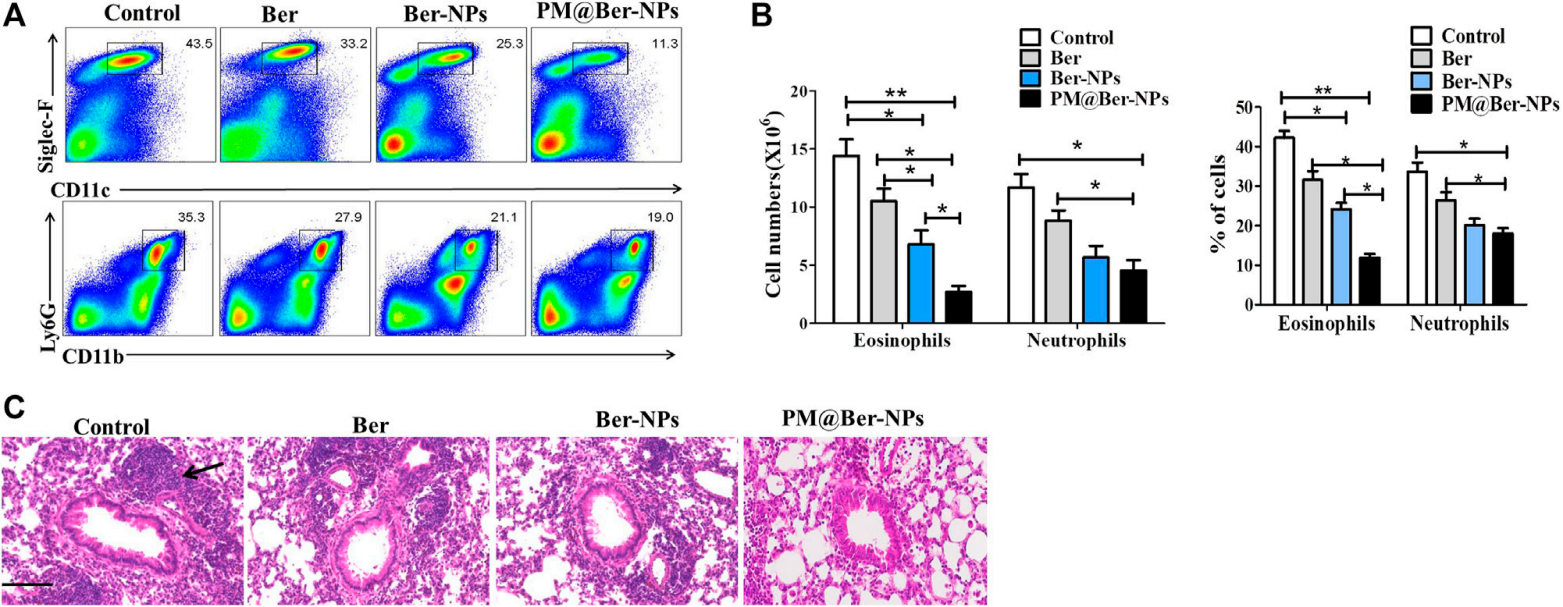
Ber-loaded biomimetic NPs alleviate lung inflammation of asthmatic mice. **(A)** Infiltrated cells in the lung with HDM-induced asthma (*n* = 5) were isolated and followed by flow cytometry analysis. **(B)** The cell numbers and percent of different infiltrated cells were calculated for each mouse. **(C)** Hematoxylin and eosin (H&E) staining of a lung section from mice with HDM-induced asthma. Arrow shows infiltrated cells from blood vessels accumulated around mice’s lung airway. Scale bars, 100 μm. *p < 0.05; **p < 0.01.

To further examine the effect of Ber and its NPs on the HDM-induced asthma model, we isolated infiltrated cells in lungs from asthmatic mice. Data by flow cytometry showed that the cell numbers and percentage of eosinophil and neutrophil were decreased by Ber and its NPs (Figures 4A,B). Moreover, the cell numbers and percentages of infiltrated cells in the lung by PM@Ber-NPs have significant differences compared to other groups. H&E staining also showed that Ber and its NPs reduced leukocytes infiltration in mouse airway of the lung (Figure 4C). These results are consistent with the infiltrated cell numbers in BALF. Combined with all data together, it suggests that Ber and its NPs could alleviate lung inflammation. Among them, PM@Ber-NPs exhibited the best beneficial effects to reduce airway inflammation in HDM-induced asthma.

### Ber-Loaded Biomimetic NPs Reduce Airway Inflammation of Mice *via* Enhancing IL-12 Expression

**FIGURE 5:**
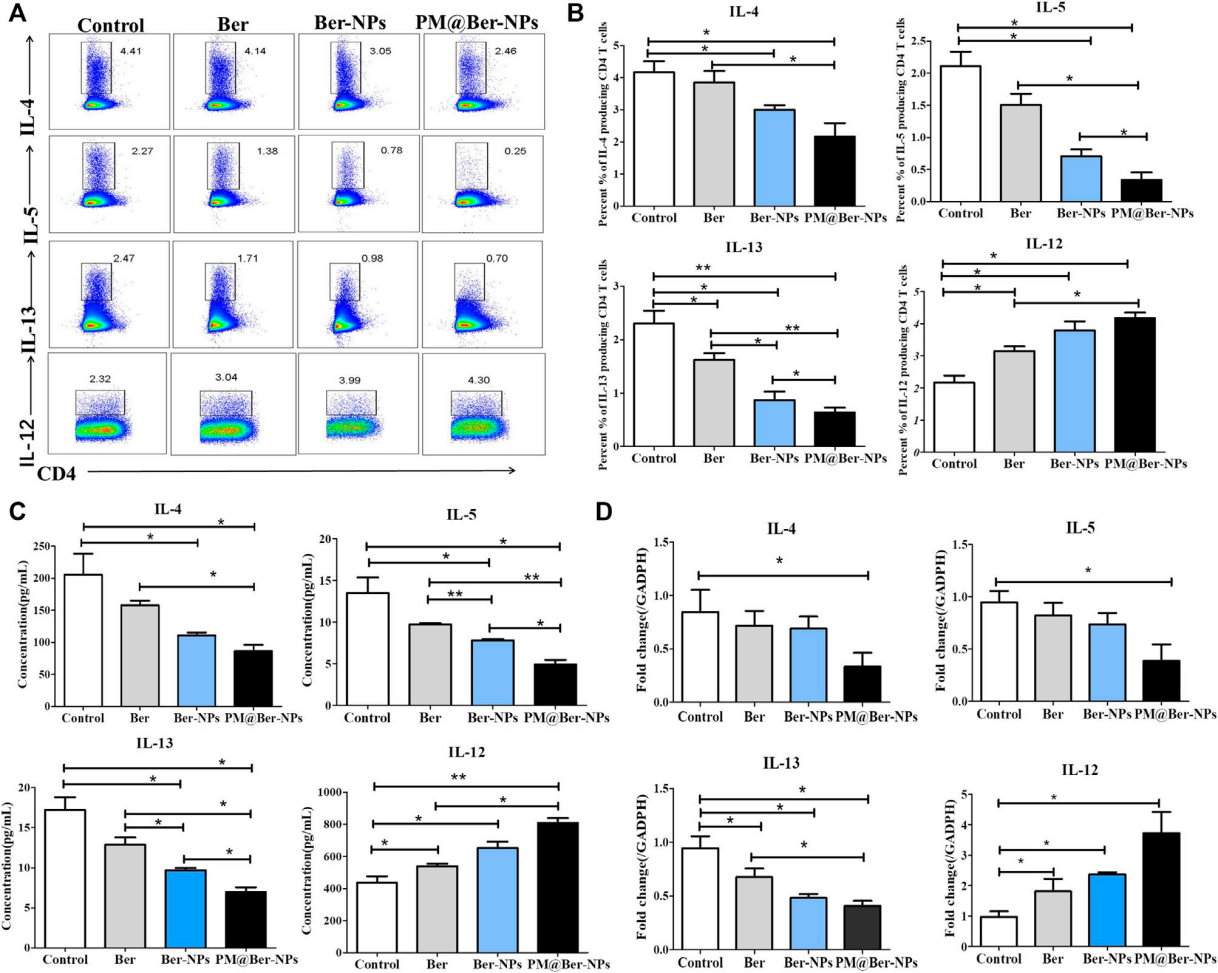
Ber-loaded biomimetic NPs decrease Th2 type cytokines expression but enhance IL-12 expression in lungs of asthmatic mice. **(A)** Cytokines secretory in the lungs from HDM-induced asthma mice (*n* = 5) were isolated and followed by flow cytometry analysis. **(B)** Percentages of cytokines secretory in the lungs from HDM-induced asthma mice were counted. **(C)** Th2 type cytokines (IL-4, IL-5, and IL-13) and IL-12 serum from asthmatic mice were determined by ELISA kit. **(D)** The mRNA expression of cytokines was examined by real-time RT-PCR. *p < 0.05; **p < 0.01.

To understand the mechanisms involved in inhibiting airway inflammation by Ber, we examined the expressions of cytokines by flow cytometry in asthmatic mice. The data showed that Ber and its NPs inhibited Th2 type cytokine expression (Figures 5A,B) but increased the IL-12 expression (Figures 5A,B). We also examined the Th2 type cytokines and IL-12 secretion in asthmatic mice’s sera. The results showed that Ber and its NPs reduced Th2 type cytokine secretion but increased IL-12 secretion (Figure 5C). qPCR also showed that Ber and its NPs reduced IL-13 mRNA expression (Figure 5D). PM@Ber-NPs significantly suppressed IL-4, IL-5, and IL-13 expression and exhibited higher inhibitory effects. In addition, Ber and its NPs increased IL-12 expression (Figures 5A–D). These results indicated that Ber modulates Th1/Th2 balance. Thereby, Ber and its NPs suppressed airway inflammation through enhancing IL-12 expression.

## Discussion

Chinese herbal medicine treatment of chronic asthma has a long history in China and around the world (Ouyang et al., 2020; Wong et al., 2021). It has been reported that Ber has various activities including anti-inflammatory effects and has been used in treating many diseases including asthma (Yang et al., 2014; Li et al., 2016; Tew et al., 2020). However, the low bioavailability of Ber limits its clinical application as an anti-inflammatory agent. Moreover, Ber also presents other drawbacks such as poor oral absorption and first-pass effect in the intestine and liver (Liu et al., 2010). It remains in the tissue for a short time after 24 h because of drug metabolization and clearance. To overcome these issues, the development of targeted drug delivery based on nanotechnology presents a new approach for the loading and delivery of Ber to improve its bioavailability (Wu et al., 2014; Mirhadi Rezaee, et al., 2018; Li et al., 2019; Lu et al., 2019; Niu et al., 2020). But Ber based on nanotechnology in asthma treatment has not been reported until now. Thereby, in this study, we tried to develop a new Ber based on nanotechnology to treat asthma.

Biomimetic cell membrane-coated NPs have been broadly applied because of their superior biochemical properties. Cell membrane-coated NPs have been demonstrated to create a platform for a variety of applications, including detoxification (Hu et al., 2013a; Pang et al., 2015), drug delivery (Hu et al., 2011), and vaccination (Hu et al., 2013b; Fang et al., 2014). Functionalization with PM enables biomimetic targeting by taking advantage of the natural interactions between platelet surface markers and different targets, including damaged airway and pathogens (Hu et al., 2015a; Hu et al., 2015b). Given the wide range of biological interactions that PM participates in, the potential of PM-coated NPs extends far beyond the traditional nanodelivery applications. The reason might be that the biodetoxification of PM coated with NPs serves as an ideal substrate for interaction with biological toxins, enabling their neutralization and subsequent clearance. Recently reported papers have shown that PM coated with NPs has a good target for treatment of lung diseases (Bahmani et al., 2021; Zhou et al., 2021). However, PM coated with NPs for targeted treatment of asthma still was little known.

In the present study, *in vitro* results showed that PM@Ber-NPs exhibited enhanced cellular uptake in the inflammatory microenvironment compared with free Ber and Ber-NPs. Moreover, *in vivo* fluorescence imaging revealed that more PM@Ber-NPs were targeted to lung tissues than free Ber and Ber-NPs. In a mouse model of HDM-induced asthma, the PM@Ber-NPs markedly inhibited lung inflammation as evidenced by the reduced number of inflammatory cells and the expression of inflammatory cytokines in the lung compared with free Ber and Ber-NPs treatments. In addition, in this study, the administrated dose of Ber-loaded NPs was only 2 mg/kg body weight, which is lower than that of previous studies (Li et al., 2016). These results suggested that delivery of PM@Ber-NPs has improved its bioavailability than free Ber and Ber-NPs.

It has been reported that imbalanced Th1/Th2 cells exist in patients with allergic asthma (Shi et al., 2011). The alternation of the Th1/Th2 ratio is confirmed to be an initial factor for asthma that increases airway inflammation (Carneiro et al., 2010). Therefore, it could be a potentially beneficial remedy for asthma treatment if the imbalanced status of Th1/Th2 cells can be reversed. To further investigate the mechanism of Ber in asthma, we detected cytokines expression associated with Th1 and Th2 cells by flow cytometry and ELISA. Our findings showed that Ber markedly inhibited expressions of Th2 cytokines (including IL-4, IL-5, and IL-13) and elevated levels of Th1 cytokines (IL-12). Allergic asthma is primarily mediated by Th2 cells, which secrete the characteristic cytokines IL-4, IL-5, and IL-13. In contrast to Th2 cells, Th1 cells have an opposite effect. IL-12 is the predominant cytokine produced by Th1 cells. IL-12 is involved in the antagonism of Th2-cell responses and IgE synthesis to restrain the progress of asthma. Accordingly, examination of the levels of Th1/Th2 cytokines is an important index in the evaluation of asthma. To further confirm the effects of Ber on Th1/Th2 cytokines, we determined the mRNA expressions of IL-12, IL-4, IL-5, and IL-13 in lung tissues. As expected, the result of real-time PCR is consistent with that of flow cytometry and ELISA. This result suggests that Ber could be able to regulate the immune response of Th1/Th2 cells. Because of the increased IL-12 expression in asthma, Ber inhibits airway inflammation in asthmatic mice. These roles of Ber in other anti-inflammation diseases have similarly been shown (Kim et al., 2003).

In conclusion, Ber encapsulated into biomimetic PLGA NPs indicated an unbalanced Th1/Th2 response for effective delivery of drugs to the inflammatory tissue in the asthma model. PM@Ber-NPs were prepared and characterized, which ameliorate HDM-induced asthma. The novel drug nanodelivery system may provide a promising platform for improving asthma treatment.

## Data Availability

The original contributions presented in the study are included in the article/Supplementary Material; further inquiries can be directed to the corresponding author.

## Supplementary Material

Supplementary Figure S1 The drugs accumulate in murine inflammatory lungs, but not in the brain *via* biomimetic modification of NPs with PM. Cou6 was encapsulated into the NPs as a fluorescence marker. **(A)** Biodistribution and retention of PM@blank-NPs, naked Cou6-NPs without modification, and PM@Cou6-NPs in organs, determined by IVIS imaging system at 2 h after NP intranasal administration. **(B)** The mean fluorescence intensity (MFI) of NPs in lungs, determined by the IVIS imaging system. * *p* < 0.05; ** *p* < 0.01.
