# Supplementary figures and images for: Berberine-Loaded Biomimetic Nanoparticles Attenuate Inflammation of Experimental Allergic Asthma via Enhancing IL-12 Expression
Source: Front Pharmacol. 2021 Nov 9;12:724525. doi: 10.3389/fphar.2021.724525 (PMC8630696; doi:10.3389/fphar.2021.724525)

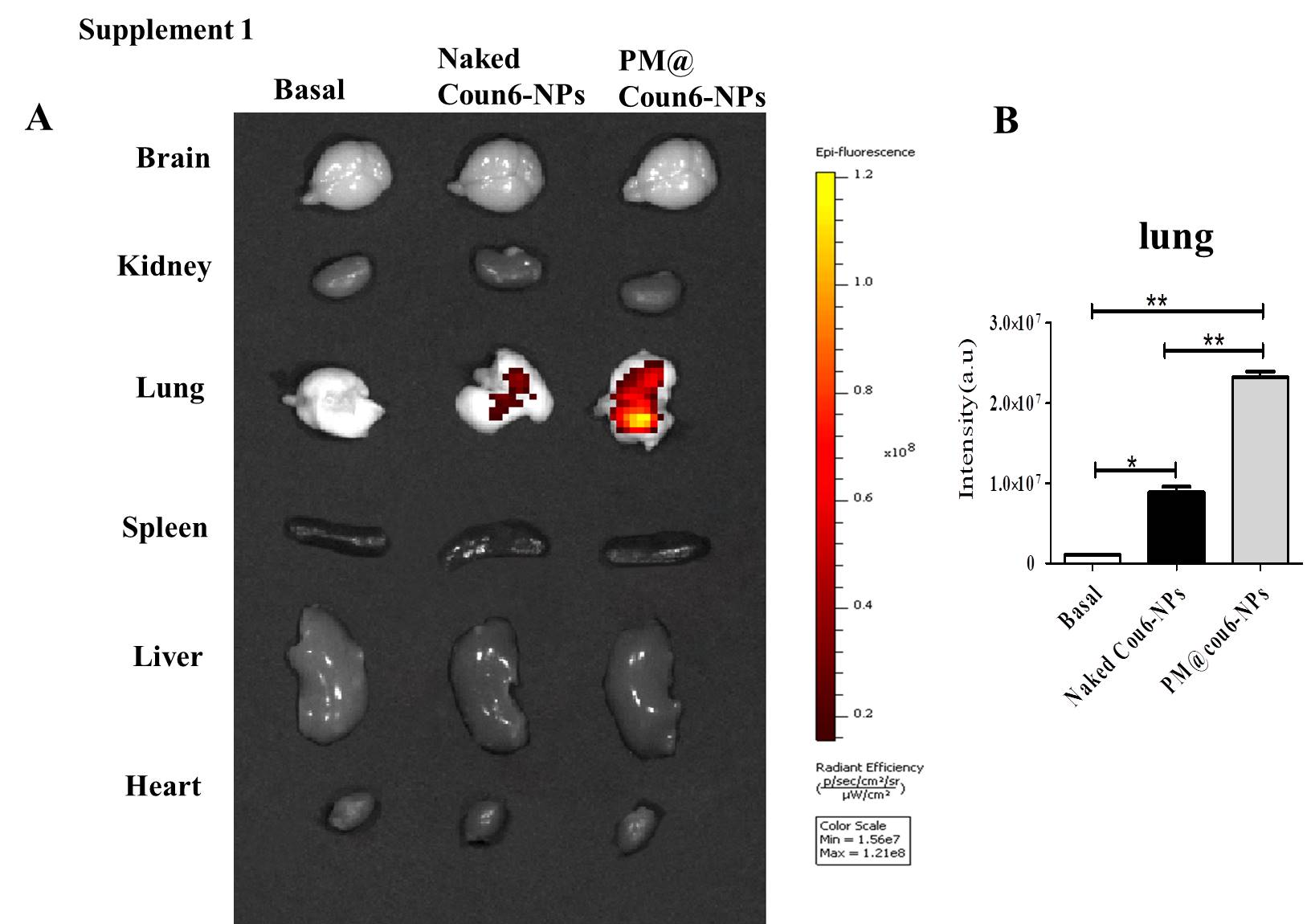

Supplement: Supplementary file 1 [file Image1.jpg]
